# Syndromic Retinitis Pigmentosa: A 15-Patient Study

**DOI:** 10.3390/genes15040516

**Published:** 2024-04-20

**Authors:** Ianne Pessoa Holanda, Priscila Hae Hyun Rim, Rare Genomes Project Consortium, Mara Sanches Guaragna, Vera Lúcia Gil-da-Silva-Lopes, Carlos Eduardo Steiner

**Affiliations:** 1 Genética Médica e Medicina Genômica, Departamento de Medicina Translacional, Faculdade de Ciências Médicas, Universidade Estadual de Campinas (Unicamp), Campinas 13083-888, SP, Brazil; ianne@unicamp.br (I.P.H.); mrsanche@unicamp.br (M.S.G.); vgslopes@unicamp.br (V.L.G.-d.-S.-L.); 2 Ambulatório de Genética Ocular, Departamento de Oftalmologia e Otorrinolaringologia, Faculdade de Ciências Médicas, Universidade Estadual de Campinas (Unicamp), Campinas 13083-888, SP, Brazil; priscilarim@gmail.com; 3 Serviço de Genética Molecular, Departamento de Medicina Laboratorial, Hospital Israelita Albert Einstein (HIAE), São Paulo 05652-900, SP, Brazil

**Keywords:** molecular diagnoses, retinitis pigmentosa, genetic testing, Bardet–Biedl syndrome, Usher syndrome, precision medicine

## Abstract

Retinitis pigmentosa is a group of genetically determined retinal dystrophies characterized by primary photoreceptor apoptosis and can occur in isolated or syndromic conditions. This study reviewed the clinical data of 15 patients with syndromic retinitis pigmentosa from a Rare Disease Reference Center in Brazil and the results of their next-generation sequencing tests. Five males and ten females participated, with the mean ages for ocular disease onset, fundoscopic diagnosis, and molecular evaluation being 9, 19, and 29 years, respectively. Bardet–Biedl syndrome (*n* = 5) and Usher syndrome (*n* = 3) were the most frequent diagnoses, followed by other rare conditions. Among the patients, fourteen completed molecular studies, with three negative results and eleven revealing findings in known genes, including novel variants in *MKKS* (c.432_435del, p.Phe144Leufs*14), *USH2A* (c.(7301+1_7302-1)_(9369+1_9370-1)del), and *CEP250* (c.5383dup, p.Glu1795Glyfs*13, and c.5050del, p.Asp1684Thrfs*9). Except for Kearn-Sayre, all presented an autosomal recessive inheritance pattern with 64% homozygosity results. The long gap between symptom onset and diagnosis highlights the diagnostic challenges faced by the patients. This study reaffirms the clinical heterogeneity of syndromic retinitis pigmentosa and underscores the pivotal role of molecular analysis in advancing our understanding of these diseases.

## 1. Introduction

Retinitis pigmentosa (RP) constitutes a group of genetically determined retinal dystrophies characterized by progressive photoreceptor degeneration encompassing rods and cones [1]. Considered a neurodegenerative disease due to photoreceptor cell death and retinal pigment epithelium atrophy, it initially manifests as nyctalopia and is followed by continuous vision loss until blindness. Onset age is highly variable, and early-onset RP subtypes tend to progress rapidly, starting around ten years old. Vision impairment is evident and refractory by age 40–50 [2]. The diagnosis of RP primarily relies on fundoscopic examination, revealing characteristic features such as pigment deposits resembling bony spicules, attenuated retinal arterioles, peripheral retinal epithelial atrophy, and a pale optic disc; the extent of these findings varies depending on the disease stage [3]. This visually debilitating condition afflicts approximately 2.5 million individuals globally, disproportionately impacting individuals under 60 years of age. RP impairs the accomplishment of daily tasks and employment maintenance, causing a significant reduction in quality of life and psychological well-being [4,5].

The genetic nature of RP is well-established, with most cases demonstrating an inherited pattern [3]. Following diagnosis, it is crucial to determine whether RP occurs as an isolated condition or represents an ocular manifestation of a broader multisystemic disorder categorized as Syndromic Retinitis Pigmentosa (SRP). The four main categories of SRP include ciliopathies, Usher syndrome, inborn errors of metabolism, and mitochondrial disorders. Comprehensive management and treatment planning for individuals affected by RP necessitate a thorough understanding of these conditions.

Ciliopathies, arising from dysfunction of primary cilia critical for sensory perception, signal transduction, and development, often exhibit pleiotropic effects, impacting multiple systems beyond the eyes, such as the urinary, central nervous, and skeletal systems [6,7]. Photoreceptors, modified primary cilia themselves, are particularly susceptible to ciliopathy-related dysfunction, leading to RP as observed in Bardet–Biedl syndrome (BBS), Senior-Løken syndrome, and Alstrom syndrome [7,8].

Usher syndrome (USH) affects both the retinal stereocilia and the connecting cochlear cilium, resulting in hearing loss and, in some cases, vestibular dysfunction. This has led some to consider USH a ciliopathy [9]. However, the non-ciliary nature of USH proteins prompts others to disagree with this classification [10]. Among SRPs, USH is the most prevalent, encompassing approximately 18% of all RP cases and presenting with a combination of RP, sensorineural hearing loss, and occasional vestibular involvement [9,11]. While traditionally classified into four subtypes based on the age of onset and vestibular involvement, distinct vestibular phenotypes within these groups and the genetic accuracy of this classification are called into question [12,13,14].

Inborn errors of metabolism, another significant cause of SRP, can impact photoreceptors in diverse ways. For instance, neuronal ceroid lipofuscinoses and mucopolysaccharidosis can lead to substance accumulation within the retina [15,16]. Also, abetalipoproteinemia, causing lipid and fat-soluble vitamin E deficiencies, can result in RP [17]. Lipid metabolism disorders like Refsum disease, Sjögren-Larsson syndrome, and peroxisomal disorders like Zellweger syndrome represent further examples of metabolic conditions contributing to SRP [17,18].

Mitochondrial diseases, another leading group of SRPs, can also affect the retina due to photoreceptors’ high energy demands. Mitochondrial dysfunction can trigger photoreceptor apoptosis and RP, as seen in MELAS, Kearn-Sayre syndrome (KSS), and Leigh syndrome [17].

Acknowledging the diverse nature of SRP and recognizing the existence of additional syndromes beyond those mentioned is essential. The critical step lies in establishing a specific diagnosis, encompassing both clinical and molecular aspects, for each case of SRP to ensure optimal care coordination and effective genetic counseling.

The Brazilian population is extensively mixed, and its genetic background is mainly descended from three major ethnic populations: Europeans, Africans, and Native Americans [19]. Additionally, within the Brazilian population, the occurrence rates of inherited retinal dystrophies are underreported, and their underlying genes are under-investigated. Nonetheless, the proportional rates of various inherited retinal dystrophies in Brazil appear to be similar to those observed globally, with non-syndromic retinitis pigmentosa, Stargardt disease, and Leber congenital amaurosis prevailing as the most common dystrophies, followed by syndromic inherited retinal dystrophies [20].

## 2. Patients and Methods

This case series aimed to analyze clinical and molecular data from patients with SRP. Patients with confirmed or suspected syndromic conditions featuring the fundoscopic diagnosis of RP who attended a single Reference Center for Rare Disease in Campinas, Brazil, from 2019 to 2023 were recruited. Individuals were invited to join a research protocol approved by the Institutional Ethics Committee, and written consent was obtained from the patients or legal guardians before procedures. Data collection comprised the information presented in Supplementary File S1; all data was obtained from medical records and confirmed by patients and relatives. Furthermore, molecular results of previously performed new-generation sequencing (NGS) tests were analyzed, specifically gene panel sequencing (GPS) for retinopathies, whole exome sequencing (WES), or whole-genome sequencing (WGS).

GPS was performed on DNA extracted from peripheral blood samples. These samples were enriched for targeted regions using a hybridization-based protocol and sequenced using Illumina technology. The gene panel consisted of sequence analysis and copy number variation (CNV) testing for 328 genes (Supplementary Material), performed by Invitae Corporation. Targeted regions were sequenced with a minimum depth of 50×, and the reference genome was GRCh37/hg37.

WES was performed at Mendelics Genomic Analysis (São Paulo, Brazil) facilities using Illumina NovaSeq 6000. The sequencing library was built with Illumina Nextera Flex, and a Customized Exome Kit from Twist Biosciences was used to capture target regions. Sequencing of samples results in paired 101 bp sequences mapped to the hg38 reference using BWA MEM software version 1.1.4 (http://bio-bwa.sourceforge.net/) accessed on 12 March 2024. The resulting VCF files were processed using Mendelics’ in-house pipeline for annotation and filtering. Quality metrics were minimum coverage over 10× and at least 90% depth.

WGS was performed on DNA extracted from peripheral blood using an Illumina platform, following mechanical fragmentation and a PCR-free protocol. Data were processed to detect point mutations, CNVs, and structural variants according to best practices for bioinformatics pipelines [21]. Quality metrics were a minimum coverage of 20× and at least 90% depth above 15×. The reference genome was GRCh38/hg38.

All the NGS reports (GPS, WES, and WGS) provided a classification of variants. However, we reclassified the variants according to the American College of Medical Genetics (ACMG) criteria recommendations to standardize variant classification, using the refinements proposed by the Sequence Variant Interpretation Working Group by 2023 [22,23]. Our group followed the nomenclature recommendations of the Human Genome Variation Society [24]. A variant was considered novel if it had not been previously published or registered in the Clinvar database [25]. When a variant was present in Clinvar in only one assertion, and that assertion corresponded to one of our patients, we also considered it a novel variant.

Additionally, low-pass whole genome sequencing (LP-WGS) was performed in two patients using the Illumina DNA PCR-Free Library Prep tagmentation protocol (Illumina, San Diego, CA, USA) and quantified by qPCR assay using the KAPA Library Quantification Kit (Roche, Switzerland). Experiments were performed on the NovaSeq 6000 equipment (Illumina, USA) using the NovaSeq 6000 S4 Reagent Kit v1.5 (300 cycles) in a paired-read sequencing strategy with 150 cycles from each end of the DNA fragment. CNV data analysis was performed using the NxClinical software 5.0 (BioDiscovery, El Segundo, CA, USA).

## 3. Results

Fifteen patients from fourteen unrelated families with syndromic conditions featuring a fundoscopic retinitis pigmentosa diagnosis were enrolled. The sex ratio was 1:2 (M:F). Consanguinity was present in four (31%) families, and familial recurrence was present in three (23%) families, all in the sibship. The median age at the onset of vision impairment and fundoscopic diagnosis was 9 and 19 years, respectively, with a median gap of ten years.

Regarding syndromic diagnosis, five patients had BBS, three USH, two with *AGBL5*-associated phenotype, one KSS, one *CEP250*-associated phenotype, and three with undiagnosed syndromic conditions. Except for KSS, which presents mitochondrial inheritance, all the other syndromic diagnoses follow autosomal recessive inheritance.

Excluding patient BB5, all other patients diagnosed with BBS were initially identified based on clinical criteria.

Concerning USH, three patients were diagnosed with the combination of RP and SNHL; none of them presented vestibular dysfunction signals. Patient US3 stood out for her multiple comorbidities.

The most common non-ocular findings among the patients were bilateral sensorineural hearing loss (SNHL) in 47% of the overall sample, while obesity and postaxial polydactyly (PAP) were seen in 33% of the overall sample, with higher frequency in the BBS group where they were seen, respectively, in 100% and 60% of the subjects.

Patient BB1, the offspring of consanguineous parents, exhibited a classical presentation of BBS and had a clinical diagnosis in early infancy. Throughout her life, she faced severe complications of the disease, including renal failure (requiring transplantation), keratoconus, and schizophrenia. Her condition was confirmed at age 27, revealing the homozygous variant c.1375C>T in the *BBS12* gene.

Patient BB2 also exhibits a classic presentation of BBS, and molecular confirmation identified compound heterozygosity for the c.271dup and the c.1122dup variants in the *BBS10* gene.

Patient BB3′s molecular evaluation showed compound heterozygosity for the c.1375C>T variant, the same presented by the BB1 patient, and the c.1627G>A variant, a nucleotide change in the *BBS12* gene.

BB4 is the son of consanguineous parents and presents the c.432_435del homozygous variant in the *MKKS* gene.

Patient BB5, daughter of consanguineous parents, presented with loss of vision at the age of twenty-four years, which was later diagnosed as RP. Her molecular evaluation revealed the homozygous variant c.1169T>G in the *BBS1* gene.

**Figure 1:**
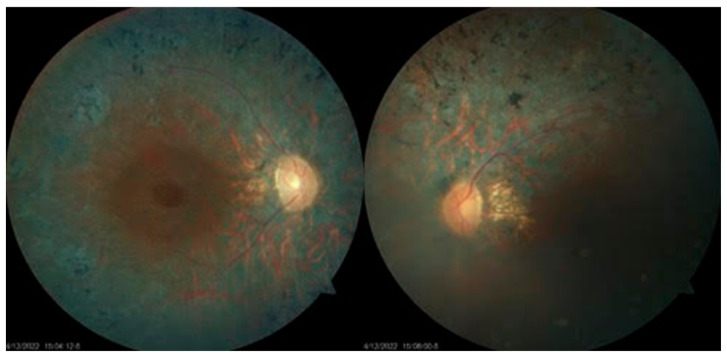
A typical fundoscopic aspect of retinitis pigmentosa in patient US1, with pigment in the form of bony spicules in 360 degrees, vascular thinning, and waxy pallor aspect of the optic disc.

Patient US1 presented with RP (Figure 1), progressing into vision impairment from early childhood and progressive hearing loss, but had no balance problems.

Patient US2, the son of non-consanguineous parents, also exhibited both hearing loss and vision impairment in early childhood, leading to a clinical classification of Usher type 2 syndrome. The NGS study revealed a homozygous c.189C>A p.(Tyr63*) variant in the *CLRN1* gene.

Patient US3 had neurosensorial hearing loss since early childhood; at the age of 28, she developed nyctalopia and peripheral vision loss, which was diagnosed as RP; due to this, a diagnosis of Usher syndrome type 2 was suspected. After WGS evaluation, two variants were found in the *USH2A* gene, c.2299del and c.14285A>G.

The present study also identified a pair of sisters, AG1 and AG2, born to first cousins’ parents, with a syndromic presentation of early childhood vision and hearing impairment, later defined as RP and neurosensory hypoacusis, respectively. NGS revealed they carried the homozygous variant c.938G>A in the *AGBL5* gene.

Another patient, CP1, presented with childhood-onset vision impairment due to RP, hearing impairment, ataxia, and vitiligo. Initially diagnosed with Usher syndrome, the WGS study revealed two variants, c.5383dup and c.5050del, in the *CEP250* gene, associated with a syndromic form of RP known as cone-rod dystrophy and sensorineural hearing loss type 2.

**Figure 2:**
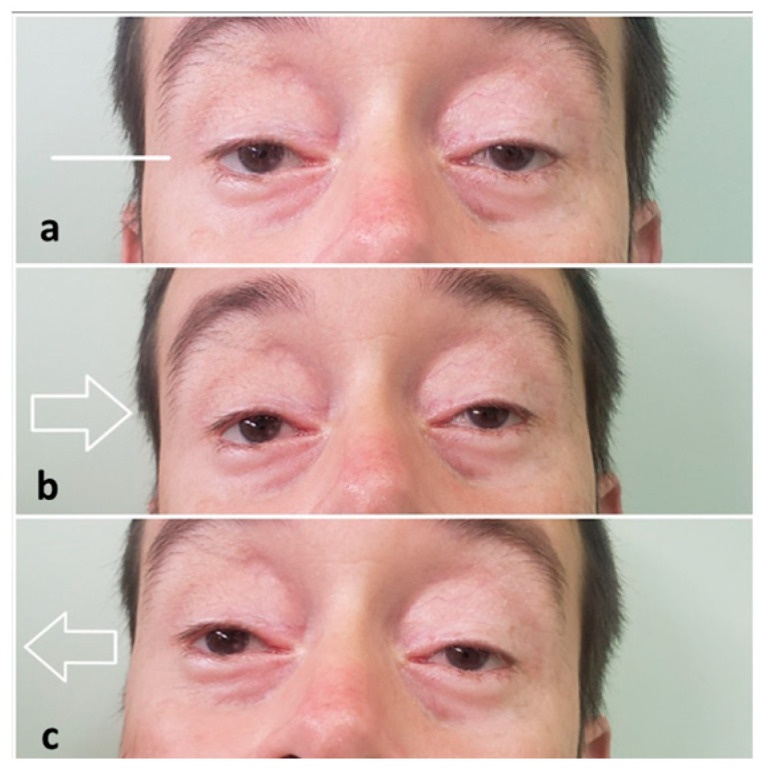
Ocular region of patient KS1 showing residual blepharoptosis after three surgical blepharoplasties, and external ophthalmoplegia when requested to look forward (**a**), to the left (**b**), and to the right (**c**).

This study also included a patient with KSS, identified as KS1. The onset of symptoms occurred at 12 years of age, manifesting as severe blepharoptosis, progressive external ophthalmoplegia, and visual impairment (Figure 2). The fundoscopic diagnosis of pigmentary retinopathy was established at 22 years. In addition to ocular findings, the patient developed progressive muscular hypotrophy, respiratory restriction, and dysarthria. A muscular biopsy supported the KSS diagnosis, but there was no diagnostic confirmation through molecular testing.

In this case series, there were three patients for whom a final diagnosis could not be established.

Patient UD1 presented with RP associated with congenital bilateral SNHL, cerebellar ataxia, and hypergonadotropic hypogonadism. The complementary investigation included chromosome microarray analysis, targeted GPS, and WGS, but no candidate variants were identified.

Patient UD2 presented with RP in the first year of life and was initially thought to have Leber congenital amaurosis due to the early onset of symptoms and significant vision impairment. However, it became later evident that the RP was part of a broader diagnosis, as she developed epilepsy (onset at seven months), microcephaly, global developmental delay, and dysphagia. UD2 underwent LP-WGS and GPS for retinopathy target genes without significant findings, as was the investigation through WES, which did not find variants related to her clinical picture.

Finally, the third undiagnosed patient is UD3, who had early-onset RP associated with a right foot post-axial polysyndactyly and global developmental delay. A ciliopathy, probably BBS, was suspected but was not confirmed in the WES. An LP-WGS test was performed and was also negative.

A summary of the clinical findings of the 15 patients is presented in Table 1.

Patients were diagnosed with RP through typical fundoscopic changes. For some of them, complementary evaluation with further exams included retinal scanning that confirmed RP fundoscopic findings, such as pale optic discs and retinal pigmentary alterations in patients AG2 and UD2. Patient BB5 underwent optical coherence tomography showing pachychoroid and thinning of outer retinal layers, fluorescein angiogram showing fibrotic macular scar, discal hyperfluorescence, and peripheral leakage, and electroretinography with confirmatory RP changes.

The present cohort’s mean age for performing NGS testing was 29 years. The genes involved were *BBS1*, *BBS10*, *BBS12*, *MKKS*, *USH2A*, *CLRN1*, *AGBL5*, and *CEP250*. Most variants are frameshift, resulting in premature stop codons, missense, and nonsense variants. Four novel variants were identified in *MKKS*, *USH2A*, and *CEP250*. Seven patients had homozygous variants. For patients BB2, BB3, US3, and CP2, it was not possible to confirm whether these variants were in trans due to limitations in testing the parents.

Table 2 shows the molecular results of this case series according to the available NGS test and ACMG criteria.

## 4. Discussion

The average interval between the onset of RP symptoms and the fundoscopic diagnosis was ten years. Such delayed diagnoses have been observed for other retinal conditions in Brazilian studies [31,32]. Late referral to the ophthalmology specialist is one hypothesis for explaining the matter; however, Oliveira and Arieta studied the flow of care in ophthalmology in the Campinas region and found that the waiting time for ophthalmology consultation is between 30 and 60 days, indicating that delay in ophthalmologist referral might not be the cause for such a long gap in RP diagnosis [33]. Moreover, two of our patients (US1 and AG2) spontaneously reported that, although they received ophthalmological evaluation shortly after experiencing vision impairment symptoms, their eye condition was managed as refractive errors, and fundoscopic evaluation took many years. This raises the hypothesis that the delay in performing fundoscopic evaluation might cause the Brazilian health system’s failure to identify and diagnose retinopathies properly. Besides, the gap between fundoscopic diagnosis and molecular testing was also ten years, reflecting the challenges the Brazilian health system faces in offering genetic testing to its population due to cost and lack of funding [34].

Concerning the molecular results in this cohort, the c.1375C>T variant in patient BB1 results in a premature translational stop signal (p.Gln459*). It is predicted to disrupt the final 252 amino acids of the BBS12 protein. Furthermore, it affects a segment of the BBS12 protein where other pathogenic variants have been previously identified [35,36].

The patient BB2 molecular study identified the c.271dup and the c.1122dup variants in the *BBS10* gene. The first variant causes a premature stop signal (p.Cys91Leufs*5), which is expected to truncate the last 633 amino acids of the BBS10 protein. This frameshift variant has been observed in individuals with Bardet–Biedl syndrome [26,37], and it has been detected across different ethnicities, suggesting it emerged in early states of the human diaspora [38] or due to a mutational hotspot. The second variant creates a premature stop signal (p.Ile375Tyrfs*3), which is expected to truncate the last 349 amino acids of the BBS10 protein. Additionally, this variant disrupts a protein region in which other variants have been determined to be pathogenic [39,40].

Patient BB3’s molecular evaluation showed compound heterozygosity for the c.1375C>T variant, the same presented by the BB1 patient, and the c.1627G>A variant, a nucleotide change in the *BBS12* gene. Also, this variant scores 0.88 in silico prediction by Revel, which corresponds with strong pathogenic evidence [41].

Patient BB4 presented the c.432_435del variant in homozygosity in the *MKKS* gene. This deletion causes a premature stop signal (p.Phe144Leufs*14) in the translation of the *MKKS* gene, which is predicted to undergo nonsense-mediated decay [42]. Considering this, it is predicted to result in a complete loss of function of the *MKKS* product and, consequently, an inability to fold a range of target proteins, resulting in the clinical manifestations of BBS [43]. Despite being a novel variant, the same protein consequence has previously been reported by the variant *MKKS* c.429_434delinsTT [44].

Patient BB5 clinical presentation consisted of RP associated with obesity, with no other relevant clinical findings or comorbidities; therefore, BBS was not previously suspected. Her molecular evaluation revealed the homozygous variant c.1169T>G in the *BBS1* gene. This nucleotide substitution, which leads to the missense consequence p.(Met390Arg), is the most common single variant for BBS, and its replication in mice was proven to cause retinopathy and ventriculomegaly [45]. Indeed, it has been reported that *BBS1* patients tend to present a milder BBS phenotype [46].

Regarding the individuals with Usher syndrome, patient US1 presented the homozygous deletion of the exons 39 to 47 of gene *USH2A*. Multiexon deletions are situations where CNV and sequence variant classifications may be applicable; in this case, it was preferred to apply ACMG sequence variant criteria adapted to single-gene copy number variants proposed by Brandt et al. [47]. This variant is predicted to cause the absence of protein, and functional studies have characterized USH2A null models, indicating increased apoptosis levels in photoreceptors and reduced visual function in zebrafish and progressive photoreceptor degeneration and moderate hearing impairment in mice [48,49]. Notably, this variant had not been previously reported in individuals affected by *USH2A*-related conditions.

Patient US2 was clinically classified as having Usher type 2 syndrome. However, molecular testing revealed that the c.189C>A variant in the *CLRN1* gene diagnosed USH type 3. This nucleotide substitution leads to the nonsense p.(Tyr63*), and it has been found in other Usher syndrome patients, being reported with an exceptionally high frequency in Gado Bravo city, in the State of Paraiba, Brazil [50], the birth city of US2’s parents. This observation suggests a possible founder effect. It is also crucial to note recent criticisms of the traditional Usher syndrome classification. Doubts have been raised about vestibular phenotypic differences in these groups [13]. Additionally, there is concern that this clinical classification may lead to the diagnosis of most deaf–blindness syndromes as though they were Usher syndrome, which could be inaccurate. In this context, a more comprehensive classification with genotypic data is necessary for Usher syndrome [12].

Patient US3 presented two variants in the *USH2A* gene, c.2299del and c.14285A>G. The first is the most common disease-causing variant in the *USH2A* gene, and it is especially recurrent in Europe and North America, probably due to European migratory movements to the New World [51]. It is also the most common causative variation for RP and is predicted to result in premature termination of the protein (p.Glu767Serfs*21), leading to nonsense-mediated decay [52]. The second variant in the *USH2A* gene, c.14285A>G, disrupts the p.Asn4762 amino acid residue, and other variants that disrupt this residue have been observed in individuals with USH2A-related conditions, suggesting that this may be a clinically significant amino acid residue [53]. In addition to the USH diagnosis, the patient has the autoimmune disorders Takayasu’s arteritis and Crohn’s disease, also diagnosed at the age of 28, and autism spectrum disorder (Asperger syndrome), recognized in infancy. The association between Usher syndrome and inflammatory bowel disease has already been described in *USH1C* patients, and it was explained by the expression of product harmony in intestinal epithelial cell microvilli, which are structurally analogous to hair cell stereocilia [54]. Usherin, a 5202 amino acid protein encoded by the *USH2A* gene, is believed to be predominantly expressed in the retina and the cochlea. Interestingly, a usherin short isoform, comprising 1546 amino acids, is widely distributed in the membranes of the small intestine and the colon. However, to the best of current knowledge, *USH2A* mutations are not suggested to induce enteropathy [48,55]. Additionally, the biallelic variants of the *USH2A* gene have been documented as candidates for autosomal recessive autism, exclusively in cases of protein-truncating variants [56].

Few patients have been reported with a phenotype associated with the *AGBL5* gene, which is classically related to isolated RP [57]. However, there has been a described case of an *AGBL5* patient who presented both RP and hearing impairment; nevertheless, in that instance, the latter was presumed secondary to maternal rubella exposure [58]. Patients AG1 and AG2 presented the homozygous variant c.938G>A in the *AGBL5* gene, a nucleotide substitution with an in silico prediction score by Revel of 0.7, indicating moderate pathogenicity [41]. However, as it was classified as a variant of uncertain significance, it cannot be definitively considered causative for the disease.

KSS is a mitochondrial DNA deletion syndrome. As extraocular muscles and the retina are high-energy-demanding, the disease’s clinical presentation consists of the classic triad of pigmentary retinopathy, progressive ophthalmoplegia, and onset before 20 years of age; moreover, the characteristic “salt and pepper” retinopathy has been detected frequently, which was not the case of KS1 [59].

Finally, patient CP1 was initially diagnosed with Usher syndrome. However, WGS revealed two variants in the *CEP250* gene, associated with retinitis pigmentosa, cone-rod dystrophy, and sensorineural hearing loss [60,61]. Both variants, c.5383dup and c.5050del, generate premature stop signals, p.Glu1795Glyfs*13 and p.Asp1684Thrfs*9, respectively, predicted to undergo nonsense-mediated decay and result in loss of function [42]. It is important to note that the disruption of *Cep250* in murine models has been functionally proven to result in severe impairment of retinal function and reduced retinal thickness [60]. This is the first time these variants have been associated with a specific phenotype.

It is also interesting to note that no record of the specific combination of clinical findings of patient UD1 was found in the literature. However, the combination of ataxia, hypergonadotropic hypogonadism, and hearing loss, which partially represent the clinical presentation of UD1 and is known as AAHH, has been described and is considered ultra-rare, with no knowledge of pathogenic mechanisms or genetic factors involved [62].

The second patient with an undiagnosed syndrome, UD2, was initially thought to have Leber congenital amaurosis (LCA) due to the early onset of symptoms and significant vision impairment. Individuals with mutations in genes associated with other syndromic eye diseases might be initially diagnosed with LCA; this can occur before the emergence of syndromic characteristics or before a more comprehensive analysis of their symptoms can be conducted [63]. However, WES sequencing has detected no variants compatible with her clinical presentation.

At least, the molecular analysis of patient UD3 showed a homozygous c.158C>A (p.Ser53*) pathogenic variant in the *PAX1* gene, associated with otofaciocervical syndrome, and another likely-pathogenic homozygous variant, c.1071T>G (pIle357Met) in the *CFI* gene, associated with susceptibility to age-related macular degeneration. Once again, none of these phenotypes was considered compatible with the patient’s clinical picture.

## 5. Conclusions

SRPs are clinically and molecularly heterogeneous conditions, and it is necessary to recognize specific diagnoses to properly manage patients. In this study, the most frequent causes of SRP were BBS and USH; other uncommon causes were identified, such as *AGBL5*-associated phenotype, KSS, *CEP250*-associated phenotype, and three with undiagnosed syndromic conditions. In their diagnostic odyssey, all patients in this series faced a long gap between the onset of symptoms, the fundoscopic diagnosis, and the molecular diagnosis, which reflects the challenges of the Brazilian healthcare system in recognizing and managing individuals with RP. Concerning molecular investigation, novel variants were found in the *MKKS*, *USH2A*, and *CEP250* genes. Furthermore, the three patients with undiagnosed syndromes illustrate the need for constant improvement in the knowledge of SRP. At least, the clinical heterogeneity of SRPs is reaffirmed, underscoring the pivotal role of molecular analysis in advancing the understanding of these diseases.

## Figures and Tables

**Table 1:** Summary of identification data, sex, family history, and ocular and clinical findings.

Group	Patient		Family History		Findings	
	ID	Sex	Consanguinity	Recurrence	Ocular	Neurological/Other
BBS	BB1	F	First cousins	-	RP, keratoconus, high myopia	Global DD, central obesity, ESRD, metabolic syndrome, hands and feet PAP, epilepsy, anxiety, schizophrenia
	BB2	F	-	-	RP	Global DD, central obesity, metabolic syndrome, hands PAP, horseshoe kidney
	BB3	M	nd	nd	RP, XT	SS, central obesity, hands and feet PAP, hypogonadism, asthma
	BB4	M	First cousins once removed	+ (one sister)	RP	LD, central obesity, metabolic syndrome, hands PAP, genital hypoplasia
	BB5	F	Double second cousins	-	RP, pachychoroid, thin retina, peripheral leakage	Obesity
USH	US1	F	-	-	RP, cataract	Bilateral SNHL (from infancy)
	US2	M	-	-	RP, cataract	Bilateral SNHL (from infancy)
	US3	F	-	-	RP	Congenital club feet, bilateral SNHL (from early childhood), Crohn’s disease, Takayasu arteritis, hidradenitis suppurativa, ASD (Asperger syndrome)
*AGBL5*	AG1	F	+ (undefined)	Sisters	RP	Bilateral SNHL (from age 32 y)
	AG2	F			RP, pale optic disk	Bilateral SNHL (from age 25 y)
*CEP250*	CP1	M	-	-	RP	Bilateral SNHL (from age 7 y), vitiligo, depression, ataxia, hypergonadotropic hypogonadism
KSS	KS1	M	-	+ (one brother)	RP, PEO, blepharoptosis	SS, muscular hypotrophy, dysarthria, restrictive lung disease
undiagnosed	UD1	F	-	-	RP, XT, nystagmus	Congenital bilateral SNHL, cerebellar ataxia, hypergonadotropic hypogonadism
	UD2	F	-	-	RP, XT, nystagmus, pale optic disk	Global DD, epilepsy, microcephaly
	UD3	F	-	-	RP, nystagmus, high myopia	Global DD, right foot PAP + syndactyly, depression

Key: +: positive; -: negative; AGBL5: *AGBL5*-associated phenotype; ASD: autism spectrum disorder; BBS: Bardet–Biedl syndrome; *CEP250*-associated phenotype; DD: developmental delay; ESRD: end-stage renal disease; F: female; KSS: Kearn-Sayre syndrome; LD: learning disabilities; M: male; nd: no data; PAP: postaxial polydactyly; PEO: progressive external ophthalmoplegia; RP: retinitis pigmentosa; SNHL: sensorineural hearing loss; SS: short stature; USH: Usher syndrome; XT: exotropia; y: years.

**Table 2:** Molecular results, according to the NGS test performed, gene/transcript, variant, zygosity, and ACMG criteria and classification.

Patient	Test	Gene	Variant(s)	Zygosity	ACMG		Ref.
		Transcript			Criteria	Classif.	
BB1	GPS	*BBS12* NM_152618	c.1375C>T p.(Gln459*)	hom	PM2, PVS1 strong, PM3 sup	LP	^a^
BB2	GPS	*BBS10* NM_024685	c.271dup p.(Cys91Leufs*5) c.1122dup p.(Ile375Tyrfs*3)	het het	PVS1 str, PM3 vs, PP4 PVS1 str, PM2 sup, PP4	P LP	[26] ^b^
BB3	WGS	*BBS12* NM_152618	c.1375C>T p.(Gln459*) c.1627G>A p.(Glu543Lys)	het het	PM2, PVS1 str, PP4 PM2, PP3, PM1, PP4	LP LP	^c^ ^d^
BB4	GPS	*MKKS* NM_018848	c.432_435del p.(Phe144Leufs*14)	hom	PM2 mod, PVS1, PM3 sup, PP4	P	Novel
BB5	GPS	*BBS1* NM_024649	c.1169T>G p.(Met390Arg)	hom	PM2 sup, PM3 very strong, PP3, PP4, PS3	P	[27]
US1	GPS	*USH2A* NM_206933	c.(7301+1_7302-1)_(9369+1_9370-1)del	hom	PM2, PVS1, PM3 sup	P	Novel
US2	GPS	*CLRN1* NM_174878	c.189C>A p.(Tyr63*)	hom	PVS1, PM2 sup, PM3, PP1	P	[28]
US3	WGS	*USH2A* NM_206933	c.2299del p.(Glu767Serfs*21) c.14285A>G p.(Asn4762Ser)	het het	PVS1, PM2, PM3 vs, PP1, PP3 PM2, PM3, PM1	P LP	[29] [30]
AG1	WGS	*AGBL5* NM_021831	c.938G>A p.(Arg313His)	hom	PM2 sup, PP3, PM3 sup	VUS	^e^
AG2	GPS						
CP1	GPS	*CEP250* NM_007186	c.5383dup p.(Glu1795Glyfs*13) c.5050del p.(Asp1684Thrfs*9)	het het	PVS1, PM2 PVS1, PM2	LP LP	Novel Novel

Key: GPS: Gene Panel Sequencing; het: heterozygous; hom: homozygous; LP: likely pathogenic; mod: moderate; P: pathogenic; str: strong; sup: support; vs: very strong; VUS: variant of uncertain significance; WGS: Whole Genome Sequencing; ClinVar accession number: ^a^ VCV000531820.6; ^b^ VCV000556154.3; ^c^ VCV001043917.2; ^d^ VCV001043917.2; ^e^ VCV001213944.3.

## Data Availability

The data supporting this study’s findings are available from the corresponding author upon reasonable request.

## Supplementary Materials

The following supporting information can be downloaded at: https://www.mdpi.com/article/10.3390/genes15040516/s1, File S1: Clinical evaluation protocol; List of genes of the Gene Panel Sequencing for Retinitis pigmentosa.
