# Supplementary material for: Syndromic Retinitis Pigmentosa: A 15-Patient Study
Source: Genes (Basel). 2024 Apr 20;15(4):516. doi: 10.3390/genes15040516 (PMC11050127; doi:10.3390/genes15040516)
Supplement: Supplementary file 1 [file genes-15-00516-s001.zip › genes-2942750-supplementary.pdf]

CLINICAL EVALUATION PROTOCOL

Personal data

Name:____ Sex:________

Birth date and place:____

History of present illness

Symptoms:____

____

____

Age of: onset:_______ fundoscopic diagnosis:______ NGS evaluation:__________

Obstetrical and perinatal background

____

____

____

Medical background

Neurodevelopment: • normal • delayed

Other neurological comorbidities:__________________________________

Infectious disease:____

Dermatological comorbidities:_____________________________________

Other ocular comorbidities:_______________________________________

Otorhinolaryngological comorbidities:_______________________________

Cardiovascular comorbidities:_____________________________________

Gastrointestinal comorbidities:____________________________________

Genitourinary comorbidities:______________________________________

Orthopedical comorbidities:____

Other:____

Familial history

Parental consanguinity: • no • yes, specify:____________________________

Recurrence of the condition:____

Other relevant conditions:____

Ethnic background:____

Physical examination

Anthropometry:____

Dysmorphological features (face, torso, and limbs): _________________________

____

Complementary exams

Fundoscopy:____

____

Other ophthalmological exams:______________________________________

____

Others:____

____

____

Supplementary material: List of genes of the Gene Panel Sequencing for Retinitis pigmentosa, in alphabetic order.

ABCA4, ABCC6, ABHD12, ACBD5, ACO2, ADAM9, ADAMTS18, ADAMTSL4, ADGRA3, ADGRV1, ADIPOR1, AGBL5, AHI1, AHR, AIPL1, ALMS1, ARHGEF18, ARL13B, ARL2BP, ARL3, ARL6, ARMC9, ARSG, ASRGL1, ATF6, ATOH7, B9D1, BBIP1, BBS1, BBS10, BBS12, BBS2, BBS4, BBS5, BBS7, BBS9, BEST1, C10orf11, C12orf65, C1QTNF5, C8orf37, CA4, CABP4, CACNA1F, CACNA2D4, CAPN5, CC2D2A, CCT2, CDH23, CDH3, CDHR1, CEP164, CEP19, CEP250, CEP290, CEP41, CEP78, CEP83, CERKL, CFAP410, CHM, CIB2, CISD2, CLCC1, CLN3, CLN5, CLN6, CLN8, CLRN1, CLUAP1, CNGA1, CNGA3, CNGB1, CNGB3, CNNM4, COL11A1, COL11A2, COL18A1, COL2A1, COL9A1, COL9A2, COL9A3, CPLANE1, CRB1, CRX, CSPP1, CTNNA1, CTSD, CWC27, CYP4V2, DHDDS, DHX32, DHX38, DNAJC17, DRAM2, DSCAML1, DTHD1, EFEMP1, ELOVL4, EMC1, ERCC6, EXOSC2, EYS, FAM161A, FBLN5, FLVCR1, FRMD7, FSCN2, FZD4, GDF6, GNAT1, GNAT2, GNB3, GNPTG, GNS, GPR143, GPR179, GPR45, GRM6, GRN, GUCA1A, GUCA1B, GUCY2D, HARS, HGSNAT, HK1, HMCN1, HMX1, IDH3A, IDH3B, IFT140, IFT172, IFT27, IFT43, IFT74, IFT80, IFT81, IFT88, IMPDH1, IMPG1, IMPG2, INPP5E, INVS, IQCB1, ITM2B, JAG1, KCNJ13, KCNV2, KIAA0586, KIAA1549, KIF11, KIF7, KIZ, KLHL7, LCA5, LRAT, LRIT3, LRP2, LRP5, LYST, LZTFL1, MAK, MAPKAPK3, MERTK, MFN2, MFRP, MFSD8, MIR204, MKKS, MKS1, MPDZ, MTPAP, MTTP, MYO7A, NAGLU, NBAS, NDP, NEK2, NEUROD1, NMNAT1, NPHP1, NPHP3, NPHP4, NR2E3, NR2F1, NRL, NYX, OAT, OCA2, OFD1, OPA1, OPA3, OPN1SW, OR2W3, OTX2, P3H2, PAX2, PAX6, PCARE, PCDH15, PCYT1A, PDE6A, PDE6B, PDE6C, PDE6D, PDE6G, PDE6H, PDZD7, PEX1, PEX10, PEX11B, PEX12, PEX13, PEX14, PEX16, PEX19, PEX2, PEX26, PEX3, PEX5, PEX6, PEX7, PHYH, PITPNM3, PLA2G5, PLK4, PNPLA6, POC1B, POC5, POMGNT1, PPT1, PRCD, PRDM13, PROM1, PRPF3, PRPF31, PRPF4, PRPF6, PRPF8, PRPH2, PRPS1, RAB28, RAX2, RBP1, RBP3, RBP4, RCBTB1, RD3, RDH11, RDH12, RDH5, REEP6, RGR, RGS9, RGS9BP, RHO, RIMS1, RLBP1, ROM1, RP1, RP1L1, RP2, RP9, RPE65, RPGRIP1, RPGRIP1L, RS1, RTN4IP1, SAG, SAMD11, SCLT1, SDCCAG8, SEMA4A, SGSH, SIX6, SLC24A1, SLC24A5, SLC45A2, SLC7A14, SNRNP200, SPATA7, SPP2, TCTN1, TCTN2, TCTN3, TEAD1, TIMM8A, TIMP3, TMED7, TMEM107, TMEM126A, TMEM138, TMEM216, TMEM231, TMEM237, TMEM67, TOPORS, TPP1, TRAF3IP1, TREX1, TRIM32, TRNT1, TRPM1, TSPAN12, TTC21B, TTC8, TTLL5, TTPA, TUB, TUBGCP4, TUBGCP6, TULP1, TYR, TYRP1, UNC119, USH1C, USH1G, USH2A, VCAN, VPS13B, WDPCP, WDR19, WDR34, WFS1, WHRN, ZNF408, ZNF423, ZNF513.
